# Political Orientations Are Correlated with Brain Structure in Young Adults

**DOI:** 10.1016/j.cub.2011.03.017

**Published:** 2011-04-26

**Authors:** Ryota Kanai, Tom Feilden, Colin Firth, Geraint Rees

**Affiliations:** 1 University College London Institute of Cognitive Neuroscience, 17 Queen Square, London WC1N 3AR, UK; 2 BBC Radio 4, Television Centre, Wood Lane, London W12 7RJ, UK; 3 Wellcome Trust Centre for Neuroimaging, University College London, 12 Queen Square, London WC1N 3BG, UK

## Abstract

Substantial differences exist in the cognitive styles of liberals and conservatives on psychological measures [1]. Variability in political attitudes reflects genetic influences and their interaction with environmental factors [2, 3]. Recent work has shown a correlation between liberalism and conflict-related activity measured by event-related potentials originating in the anterior cingulate cortex [4]. Here we show that this functional correlate of political attitudes has a counterpart in brain structure. In a large sample of young adults, we related self-reported political attitudes to gray matter volume using structural MRI. We found that greater liberalism was associated with increased gray matter volume in the anterior cingulate cortex, whereas greater conservatism was associated with increased volume of the right amygdala. These results were replicated in an independent sample of additional participants. Our findings extend previous observations that political attitudes reflect differences in self-regulatory conflict monitoring [4] and recognition of emotional faces [5] by showing that such attitudes are reflected in human brain structure. Although our data do not determine whether these regions play a causal role in the formation of political attitudes, they converge with previous work [4, 6] to suggest a possible link between brain structure and psychological mechanisms that mediate political attitudes.

## Highlights

► Political liberalism and conservatism were correlated with brain structure ► Liberalism was associated with the gray matter volume of anterior cingulate cortex ► Conservatism was associated with increased right amygdala size ► Results offer possible accounts for cognitive styles of liberals and conservatives

## Results and Discussion

For many years, psychologists and sociologists asked what kind of psychological or environmental factors influence the political orientation of individuals [1]. Although political attitudes are commonly assumed to have solely environmental causes, recent studies have begun to identify biological influences on an individual's political orientation. For example, a twin study shows that a substantial amount of the variability in political ideology reflects genetic influences [2]. Moreover, such genetic influences interact with social environment. For example, political orientation in early adulthood is influenced by an interaction between a variant of a dopamine receptor gene linked with novelty seeking and an environmental factor of friendship [3]. Here we hypothesized that these interactions between genotype, environment, and political phenotype may be reflected in the structure of the brain.

Several pioneering studies have begun examining the relationship between brain activity and political attitudes [4, 6], but none have characterized brain structure. Political attitudes are typically captured on a single-item measure in which participants self-report using a five-point scale ranging from “very liberal” to “very conservative.” Despite the simplicity of such a scale, it accurately predicts voting behaviors of individuals [7] and has been used successfully to determine genetic contributions to political orientation [3]. Psychological differences between conservatives and liberals determined in this way map onto self-regulatory processes associated with conflict monitoring. Moreover, the amplitude of event-related potentials reflecting neural activity associated with conflict monitoring in the anterior cingulate cortex (ACC) is greater for liberals compared to conservatives [4]. Thus, stronger liberalism is associated with increased sensitivity to cues for altering a habitual response pattern and with brain activity in anterior cingulate cortex. Here we explored this relationship further by examining whether political attitudes correlated not just with function but also with anatomical structure of these regions.

**Figure 1:**
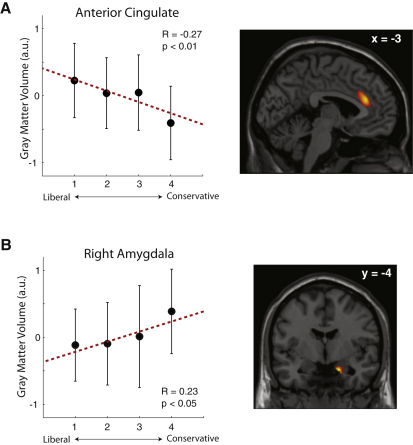
Individual Differences in Political Attitudes and Brain Structure (A) Regions of the anterior cingulate where gray matter volume showed a correlation with political attitudes (see Experimental Procedures for full details) are shown overlaid on a T1-weighted MRI anatomical image in the stereotactic space of the Montreal Neurologic Institute Template [29]. A statistical threshold of p < 0.05, corrected for multiple comparisons (see Experimental Procedures), is used for display purposes. The correlation (left) between political attitudes and gray matter volume (right) averaged across the region of interest (error bars represent 1 standard error of the mean, and the displayed correlation and p values refer to the statistical parametric map presented on the right) is shown. (B) The right amygdala also showed a significant negative correlation between political attitudes and gray matter volume. Display conventions and warnings about overinterpreting the correlational plot (left) are identical to those for (A).

To test the hypothesis that political liberalism (versus conservatism) is associated with differences in gray matter volume in anterior cingulate cortex, we recorded structural magnetic resonance imaging (MRI) scans from 90 healthy young adults (61% female) who self-reported their political attitudes confidentially on a five-point scale from “very liberal” to “very conservative” [3, 7]. We then used voxel-based morphometry (VBM) analyses [8] to investigate the relationship between these attitudes, expressed as a numeric score between one and five, and gray matter volume. We found that increased gray matter volume in the anterior cingulate cortex was significantly associated with liberalism (Figure 1A) (R = −2.71, T(88) = 2.633, p = 0.010 corrected; see Experimental Procedures for full details of analyses). We regressed out potential confounding variables of age and gender in our analysis (see Experimental Procedures). Therefore, our findings are not attributable to these factors.

Apart from the anterior cingulate cortex, other brain structures may also show patterns of neural activity that reflect political attitudes. Conservatives respond to threatening situations with more aggression than do liberals [1] and are more sensitive to threatening facial expressions [5]. This heightened sensitivity to emotional faces suggests that individuals with conservative orientation might exhibit differences in brain structures associated with emotional processing such as the amygdala. Indeed, voting behavior is reflected in amygdala responses across cultures [6]. We therefore further investigated our structural MRI data to evaluate whether there was any relationship between gray matter volume of the amygdala and political attitudes. We found that increased gray matter volume in the right amygdala was significantly associated with conservatism (Figure 1B) (R = 0.23, T(88) = −2.22, p < 0.029 corrected). No significant correlation was found in the left amygdala (R = 0.15, T(88) = −1.43, p = 0.15 corrected; see Figure S1 available online for the individual gray matter volumes of the ACC and amygdala).

Outside these regions of interest (ROIs) reflecting our prior hypotheses, we also conducted a whole-brain analysis to reveal any additional brain structures that showed correlation with political orientation. However, no regions showed such a correlation that survived correction for multiple comparisons across the whole brain (P_FWE_ > 0.05). At a more lenient statistical criterion (p < 0.001 uncorrected and cluster size larger than 50 mm^3^), we found clusters in which gray matter volume was significantly associated with conservativism in the left insula (T(88) = 4.32, R = 0.420, x = −38, y = −16, z = −2) and the right entorhinal cortex (T(88) = 3.70, R = −0.368, x = 22, y = −21, z = −26). No regions showed a positive correlation with liberalism. Thus, our data showed regional specificity for the association of political attitudes with gray matter volume in anterior cingulate and right amygdala, respectively.

To test the reliability of these findings, we next conducted a replication study using an independent sample of 28 new participants (16 female) drawn from the same demographic group (see Experimental Procedures). The procedure was identical to that described above. We replicated all the correlations between gray matter volume and self-reported political attitudes described above at all loci, including the anterior cingulate cortex (T(26) = 2.87, R = −0.491, p = 0.008), right amygdala (T(26) = −2.08, R = 0.377, p = 0.048), left insula (T(26) = −3.36, R = 0.550, p = 0.002), and right entorhinal cortex (T(26) = −3.89, R = 0.606, p = 0.0006). Thus, our findings were replicated in an independent sample of participants.

Finally, we characterized the extent to which these correlations between gray matter volume and political attitudes might permit us to determine the political attitudes of a single individual based on their structural MRI scan. We used the gray matter volume of anterior cingulate cortex and right amygdala from each individual to train a multivariate classifier [9]. A leave-one-out procedure with cross-validation was used to determine how well this classifier could predict whether an individual was conservative or very liberal when trained on the other participants' data (see Experimental Procedures for full details). The gray matter volumes of ACC and the right amygdala allowed the classifier to distinguish individuals who reported themselves as conservative from those who reported themselves as very liberal with a high accuracy (71.6% ± 4.8% correct, p = 0.011). This suggests that it is possible to determine the self-expressed political attitude of individuals, at least for the self-report measure we used, based on structural MRI scans.

Although these results suggest a link between political attitudes and brain structure, it is important to note that the neural processes implicated are likely to reflect complex processes of the formation of political attitudes rather than a direct representation of political opinions per se. The conceptualizing and reasoning associated with the expression of political opinions is not necessarily limited to structures or functions of the regions we identified but will require the involvement of more widespread brain regions implicated in abstract thoughts and reasoning.

We speculate that the association of gray matter volume of the amygdala and anterior cingulate cortex with political attitudes that we observed may reflect emotional and cognitive traits of individuals that influence their inclination to certain political orientations. For example, our findings are consistent with the proposal that political orientation is associated with psychological processes for managing fear and uncertainty [1, 10]. The amygdala has many functions, including fear processing [11]. Individuals with a large amygdala are more sensitive to fear [12], which, taken together with our findings, might suggest the testable hypothesis that individuals with larger amygdala are more inclined to integrate conservative views into their belief system. Similarly, it is striking that conservatives are more sensitive to disgust [13, 14], and the insula is involved in the feeling of disgust [15]. On the other hand, our finding of an association between anterior cingulate cortex volume and political attitudes may be linked with tolerance to uncertainty. One of the functions of the anterior cingulate cortex is to monitor uncertainty [16, 17] and conflicts [18]. Thus, it is conceivable that individuals with a larger ACC have a higher capacity to tolerate uncertainty and conflicts, allowing them to accept more liberal views. Such speculations provide a basis for theorizing about the psychological constructs (and their neural substrates) underlying political attitudes. However, it should be noted that every brain region, including those identified here, invariably participates in multiple psychological processes. It is therefore not possible to unambiguously infer from involvement of a particular brain area that a particular psychological process must be involved.

Although these conceptual links facilitate interpretations of the relationship between the brain structures and political orientation, our findings reflect a cross-sectional study of political attitudes and brain structure in a demographically relatively homogenous population of young adults. Therefore, the causal nature of such a relationship cannot be determined. Specifically, it requires a longitudinal study to determine whether the changes in brain structure that we observed lead to changes in political behavior or whether political attitudes and behavior instead result in changes of brain structure. Our findings open the way for such research. Moreover, the voting public span a much wider range of ages and demography than those studied here, and indeed political representatives themselves tend to be drawn from older adult groups. It therefore remains an open question whether our findings will generalize to these other groups or whether such demographic factors may modulate the relationship that we observed. Nevertheless, our finding that gray matter volume in anterior cingulate cortex and right amygdala can explain between-participant variability in political attitudes for young adults represents a potentially important step in providing candidate mechanisms for explaining the complex relationship between genotype, environmental factors, and political phenotype. We speculate that other aspects of political behavior may similarly have an unexpected motif in human brain structure.

Our findings show that high-level concepts of political attitudes are reflected in the structure of focal regions of the human brain. Brain structure can exhibit systematic relationships with an individual's experiences and skills [19, 20], can change after extensive training [21, 22], and is related to different aspects of conscious perception [23, 24] (see [25] for a review). We now show that such relationships with brain structure extend to complex aspects of human behavior such as political attitudes. This opens a new avenue of research to map high-level psychological features onto brain structure and to interpret sociologically motivated constructs in terms of brain functions.

## Experimental Procedures

### Participants

A total of 90 healthy volunteers (mean 23.5 ± 4.84 standard deviation [SD], 55 female) was recruited from the University College London (UCL) participant pool. Written informed consent was obtained from each participant. The study was approved by the local UCL ethics committee. We deliberately used a homogenous sample of the UCL student population to minimize differences in social and educational environment. The UK Higher Education Statistics Agency reports that 21.1% of UCL students come from a working-class background. This rate is relatively low compared to the national average of 34.8%. This suggests that the UCL students from which we recruited our participants disproportionately have a middle-class to upper-class background.

### Political Orientation Questionnaire

Participants were asked to indicate their political orientation on a five-point scale of very liberal (1), liberal (2), middle-of-the-road (3), conservative (4), and very conservative (5). This simple self-report questionnaire has been validated in a previous genetic study of political orientation [3] and is a reliable measure of political attitudes [7]. Because none of the participants reported the scale corresponding to very conservative, the analyses were conducted using the scales of 1, 2, 3, and 4.

### MRI Data Acquisition

MR images were acquired on a 1.5-T Siemens Sonata MRI scanner (Siemens Medical). High-resolution anatomical images were acquired using a T1-weighted 3D Modified Driven Equilibrium Fourier Transform sequence (repetition time = 12.24 ms; echo time = 3.56 ms; field of view = 256 × 256 mm; voxel size = 1 × 1 × 1 mm).

### VBM Preprocessing and Analysis

T1-weighted MR images were first segmented for grey matter and white matter using the segmentation tools in Statistical Parametric Mapping 8 (SPM8, http://www.fil.ion.ucl.ac.uk/spm). Subsequently, we performed diffeomorphic anatomical registration through exponentiated lie algebra in SPM8 for intersubject registration of the grey matter images [26]. To ensure that the total amount of gray matter was conserved after spatial transformation, we modulated the transformed images by the Jacobian determinants of the deformation field .The registered images were then smoothed with a Gaussian kernel of 12 mm full-width half-maximum and were then transformed to Montreal Neurological Institute stereotactic space using affine and nonlinear spatial normalization implemented in SPM8.

### ROI Analyses

A multiple-regression analysis was performed on the mean gray matter density of each ROI to determine whether they showed a correlation with the liberalism score. The total gray matter volume of individuals was included in the design matrix to regress out the general size difference across the participants.

We conducted ROI analyses on ACC and bilateral amygdala because we had prior hypotheses for these regions. The mean gray matter volume within these regions was extracted using the MarsBaR toolbox (http://marsbar.sourceforge.net/). The ROI for ACC was defined as a sphere with a radius of 20 mm centered at (x = −3, y = 33, z = 22) [4, 27]. The gray matter volume in the left and right amygdala were separately extracted using an ROI based on the Harvard-Oxford subcortical structural atlas implemented in the Oxford University Centre for Functional MRI of the Brain Software Library (http://www.fmrib.ox.ac.uk). In addition, the total gray matter volumes across the whole brain were computed from the segmented images for individual participants.

### Whole-Brain Analyses

Outside the ROIs, we conducted a whole-brain analysis. However, we did not find regions that showed significant correlations with political orientation with appropriate corrections for family-wise error (FWE) at a threshold of p < 0.05. To search for other possible candidate regions for future studies, we reported results with a slightly more lenient criterion (p < 0.001, uncorrected, cluster size >50 mm^3^; see Results and Discussion).

### Replication Study

A total of 28 healthy volunteers (mean 21.0 ± 2.5 SD, 16 female) was recruited from the UCL participant pool. Written informed consent was obtained from each to participate. The study was approved by the local UCL ethics committee. The experimental procedure and analysis was identical to that described above except that only ROI analyses were performed based on the (independent) results of the first study.

### Classification Analyses

Two-class classification between conservative and very liberal was performed using a support vector machine (SVM) algorithm [28] implemented in MATLAB and employing the gray matter volume of anterior cingulate and the right amygdala ROIs for each participant from the first main experiment (n = 90) and the replication studies. Mean classification performance was computed by leave-one-out cross-validation repeated 1000 times. The statistical significance was computed by a permutation test: the probability distribution of correct classification was estimated by running the same SVM analysis on 1000 surrogate data points created by random permutations of the labels (i.e., conservative or very liberal). The significance of the SVM performance on the original data was then estimated as the probability that the mean SVM performance on the original data was exceeded by chance (i.e., SVM performance on the permuted data).

## Supplemental Information

Document S1. One Figure
