# Supplementary material for: Political Orientations Are Correlated with Brain Structure in Young Adults
Source: Curr Biol. 2011 Apr 26;21(8):677–80. doi: 10.1016/j.cub.2011.03.017 (PMC3092984; doi:10.1016/j.cub.2011.03.017)
Supplement: Document S1. One Figure [file mmc1.pdf]

Current Biology, Volume *21*

Supplemental Information

Political Orientations Are Correlated with Brain Structure in Young Adults

Ryota Kanai, Tom Feilden, Colin Firth, and Geraint Rees

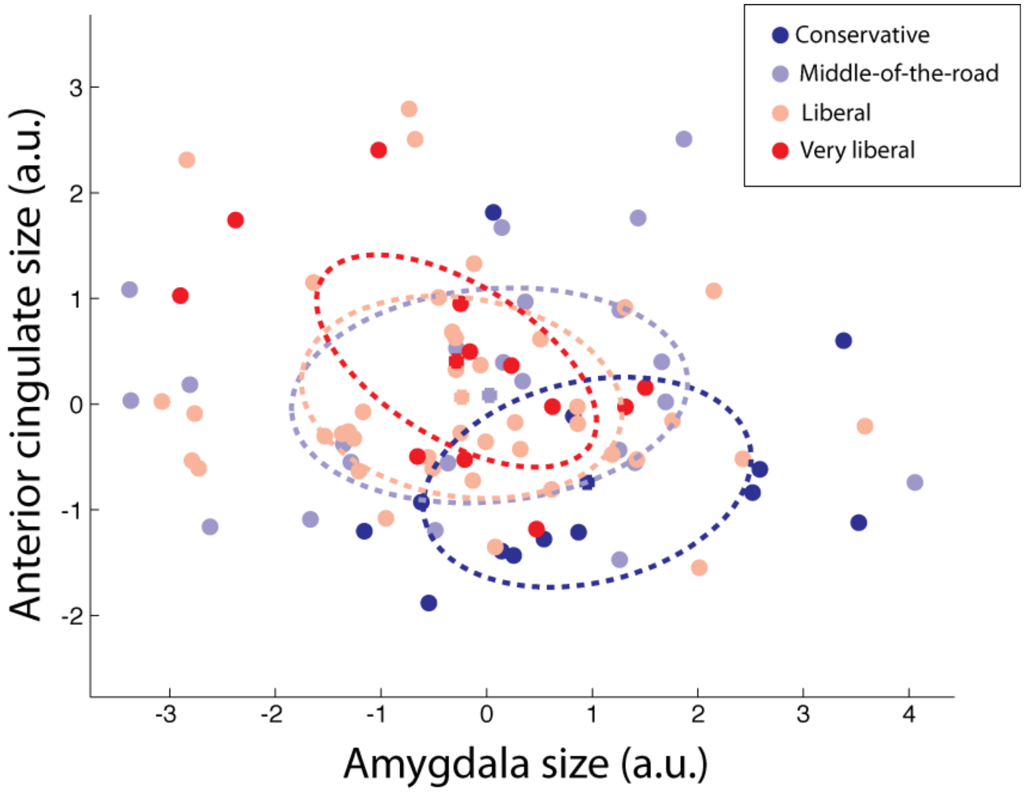

Figure S1.
Individual data (n=90) are plotted as a function of the size of the right amygdala (x-axis) and the size of anterior cingulate cortex (y-axis). The self-reported political orientation of individuals is colour-coded from blue ('conservative') to red ('very liberal'). To illustrate the inter-individual variability of brain structures for each level of political orientation, ellipses were drawn for one standard deviation along the two principal component axes.
